# An update on angiogenesis targeting in head and neck squamous cell carcinoma

**DOI:** 10.1186/s41199-020-00051-9

**Published:** 2020-04-06

**Authors:** Ida Micaily, Jennifer Johnson, Athanassios Argiris

**Affiliations:** Department of Medical Oncology, Thomas Jefferson University, 1025 Walnut Street, Suite 700, Philadelphia, PA USA

**Keywords:** Angiogenesis, HNSCC, Bevacizumab, VEGF, VEGFR, EGFR, TKI, Monoclonal antibodies

## Abstract

Angiogenesis is an integral aspect of the growth and proliferation of solid tumors, including head and neck squamous cell carcinoma (HNSCC), and has potential implications in prognosis and treatment of both localized and recurrent/metastatic HNSCC. Therefore, there has been a significant interest in utilizing anti-angiogenic agents either alone or in combination with currently approved and emerging therapies. A phase III randomized trial (E1305) of chemotherapy with or without bevacizumab in the first-line treatment of recurrent/metastatic HNSCC showed an increased response rate and longer progression-free survival but fell short in demonstrating a statistically significant improved survival with bevacizumab. Moreover, toxicity, especially bleeding, was increased. Nevertheless, the study of other anti-angiogenic agents and novel combinations with other therapies, including immunotherapy, remains of interest. Several clinical trials are currently underway.

## Introduction

Tumor growth, proliferation, and metastasis require the process of angiogenesis for nutrient delivery and growth. The formation of new blood vessels can be a rate-limiting step in the development of solid tumors. Without a critical amount of new blood vessel formation known as the angiogenic switch, tumors are unable to grow past a few millimeters in size. Therefore, angiogenesis is an attractive target in cancer therapy across multiple tumor types. Clinical benefit with anti-angiogenics has been modest, but interest in angiogenesis as a therapeutic target remains [1, 2].

### Targeting angiogenesis in solid tumor malignancies

Angiogenesis is a complex and highly regulated phenomenon that is co-opted by solid tumor malignancies. Regulation within the tumor microenvironment includes activation of receptor tyrosine kinases such as the vascular endothelial growth factor receptor (VEGFR), epidermal growth factor receptor (EGFR), fibroblast growth factor receptor (FGFR), and platelet-derived growth factor receptors (PDGFR) [3]. Preclinical data has demonstrated that VEGF contributes to immune suppression in multiple ways: by binding to VEGFR1 on myeloid derived stem cells and preventing their differentiation into mature immune cells, by inducing programmed death-ligand 1 (PD-L1) expression on dendritic cells which leads to decreased T cell activation, by decreasing T cell adhesion and extravasation through vessel walls, and by increasing Treg differentiation [4–7]. While the majority of research in anti-angiogenics has been directed towards VEGF, other pathways play a critical role that may be exploited clinically. Therefore, combining anti-angiogenics with immune checkpoint inhibitors is of clinical interest in many tumor types. Anti-angiogenic strategies are already the standard of care in a variety of malignancies including adenocarcinoma of the colon, non-squamous non-small cell lung cancer (NSCLC), renal cell carcinoma, cervical cancer, glioblastoma multiforme, ovarian cancer, hepatocellular carcinoma, soft tissue sarcoma, and gastric cancer. As of yet, there are no FDA-approved anti-angiogenics in HNSCC.

### Rationale for targeting angiogenesis in HNSCC

The interest in expanding the role of anti-angiogenics into HNSCC is rationally based. Up to 90% of HNSCC highly express angiogenesis factors like VEGF. High expression is correlated with more advanced disease, resistance to traditional cytotoxic agents, and poor prognosis [8–10]. Similarly, EGFR has been shown to be upregulated in 70–100% of HNSCC and is also correlated with poor prognosis [11, 12]. Preclinical models have provided evidence to support the targeting of angiogenic pathways in HNSCC [13, 14].

### Overview of anti-angiogenic agents

There are currently 14 different FDA-approved anti-angiogenic therapies that fall into 4 categories based on mechanism of action: ligand-directed antibodies, receptor-directed antibodies, small molecule inhibitors, and immunomodulatory agents, though this class has been relegated to the treatment of multiple myeloma.

The first-in-class ligand-directed anti-angiogenic drug is bevacizumab, a monoclonal antibody targeting VEGF-A. It was initially approved in 2004 for the treatment of colorectal cancer and has been approved to date in the treatment of 6 different solid tumors (colorectal cancer, NSCLC, glioblastoma, kidney cancer, ovarian cancer and cervical cancer). Ziv-aflibercept, a recombinant fusion protein composed of the VEGF-binding portion of VEGFR- 1 and − 2 acts as a ligand trap, preventing binding and activation of the intended VEGFR targets. This agent has been approved only in combination with chemotherapy for colorectal cancer. Dalantercept is an experimental agent comprised of activin receptor-like kinase 1 fused to a human Fc domain (ALK1-Fc) that binds to ligands such as BMP9 and 10. When bound to its ligands, ALK1-mediated angiogenesis is inhibited.

The first-in-class receptor-directed drug is ramucirumab, a monoclonal antibody targeting VEGFR2. This agent has been developed for the treatment of multiple solid tumors and has gained regulatory approval in NSCLC, gastric cancer, colorectal cancer, and hepatocellular carcinoma.

In contrast to the monoclonal antibodies, tyrosine kinase inhibitors (TKIs) are small molecules that can function by inhibiting multiple targets within angiogenic signaling pathways, including VEGFRs, EGFR, FGFR, and PDGFRs. Examples of TKIs that are utilized for their inhibition of multiple angiogenic receptors include sorafenib, sunitinib, vandetanib, pazopanib, axitinib, regorafenib, lenvatinib, cabozantinib (Table 1). While there is great interest in using VEGFR TKIs in HNSCC, none of these agents have been tested in phase III trials in HNSCC yet.

**Table 1:** FDA approved anti-angiogenic agents for the treatment of solid tumors

AGENT	DRUG CLASS	MOLECULAR TARGETS
Bevacizumab	Monoclonal Antibody	VEGF
Ramucirumab	Monoclonal Antibody	VEGFR2
Ziv-Aflibercept	Fusion Protein	VEGF, VEGF-B, PlGF
Sorafenib	TKI	RAF/MEK/ERK, VEGFR 1–3, PDGFR- β, c-KIT, FLT3, RET
Sunitinib	TKI	VEGFR1 and 2, PDGFR-α and -β, c-KIT, RET, CSF1R, FLT3
Vandetanib	TKI	VEGFR2 and 3, EGFR, RET
Pazopanib	TKI	VEGFR 1–3, PDGFR-α and -β, FGFR-1 and − 3, c-KIT
Axitinib	TKI	VEGFR 1–3, PDGFR-α and -β, c-KIT
Regorafenib	TKI	VEGFR1–3
Lenvatinib	TKI	VEGR1–3, FGFR1–4, PDGFR- α, c-KIT, RET
Cabozatinib	TKI	VEGFR2, AXL, RET, MET, c-KIT, FLT-3

*TKI* tyrosine kinase inhibitor

Development of these antibodies and TKIs in HNSCC has occurred as monotherapy as well as through combinations with other modalities and therapeutic agents: chemotherapy, radiotherapy, molecularly targeted therapy, and more recently immunotherapy.

### TKIs and other agents

TKIs have demonstrated preclinical activity against angiogenesis through multiple receptors, however these agents have yielded varying degrees of success when used for the treatment of patients with recurrent/metastatic HNSCC (Table 2) [15–23]. Sorafenib is a multi-kinase inhibitor that has been evaluated as a single-agent in recurrent/metastatic HNSCC. Phase II data showed a low likelihood of response to sorafenib in this setting [15, 16]. Sunitinib, a multi-TKI with activity against PDGFRs and VEGFRs showed similarly minimal response rates in phase II studies [17–19]. Bleeding complications and skin ulceration/fistula formation were seen in some of these studies [16, 17]. A phase II trial of axitinib demonstrated a low objective response rate but favorable disease control rate of 77% and median overall survival (OS) of 10.9 months with acceptable toxicity profile [20]. Combinations of TKIs with chemotherapy as well as cetuximab have also been studied in two randomized phase II trials without encouraging results [21, 22]. A notable exception was a single arm phase II trial of carboplatin, paclitaxel and sorafenib that reported promising efficacy with this combination regimen with a response rate of 55%, median progression-free survival (PFS) of 8.5 months and median OS of 22.6 months [23]. No randomized trials of chemotherapy with or without sorafenib have been conducted so far in HNSCC.

**Table 2:** VEGFR tyrosine-kinase inhibitors studied as monotherapy or combination therapy in HNSCC

AGENTS	STUDY DESIGN/PHASE	# of PATIENTS	PRIMARY ENDPOINT/OTHER EFFICACY ENDPOINTS	REFERENCE
**Sorafenib**				
Sorafenib 400 mg twice daily	Single arm, Phase II Single arm, Phase II	*n* = 41 *n* = 27	RR 2%; median PFS 4 mo; median OS 9 mo RR 4%; median PFS 1.8 mo; median OS 4.2 mo	Williamson, 2010 [15] Elser, 2007 [16]
**Sunitinib**				
Sunitinib 37.5 mg daily	Single arm, Phase II	*n* = 38	Rate of disease control 50%; RR 3%; median PFS 2 mo; median OS 3.4 mo	Machiels, 2010 [17]
Sunitinib 50 mg daily for 4 weeks on, 2 weeks off	Single arm, Phase II (two cohorts, cohort A: PS 0–1 cohort B: PS 2)	*n* = 22	Cohort A: RR 8%; median TTP 2 mo; median OS 4.9 mo Cohort B: RR 0; median TTP 2.5 mo; median OS 4.5 mo	Choong, 2010 [18]
Sunitinib 50 mg daily for 4 weeks on, 2 weeks off	Single arm, Phase II	*n* = 17	RR 0; median TTP 2.3 mo; median OS 4 mo	Fountzilas, 2010 [19]
**Axitinib**				
Axitinib 5 mg twice daily	Single arm, Phase II	*n* = 30	6-month PFS 30%; RR 7%; median PFS 3.7 months; median OS 10.9 mo	Swiecicki, 2015 [20]
**Combination Therapy**				
Cetuximab +/− sorafenib	Randomized, Phase II	*n* = 55	Median PFS 3 mo (cetuximab arm) and 3.2 mo (combination arm)	Gilbert, 2015 [21]
Docetaxel +/− vandetanib	Randomized, Phase II	*n* = 29	RR 7% (docetaxel arm) and 13% (combination arm)	Limaye, 2013 [22]
Carboplatin, paclitaxel, sorafenib	Single arm, Phase II	*n* = 48	Median PFS 8.5 mo; RR 55%; median OS 22.6 mo	Blumenschein, 2012 [23]

*RR* overall response rate, *PFS* progression-free survival, *TTP* time to progression, *PS* performance status, *mo* months

Dalantercept, an ALK1-Fc trap, was evaluated in a phase II monotherapy study in recurrent/metastatic HNSCC. In 40 evaluable patients the median PFS was 1.4 months and the median OS 7.1 months. While the drug was well tolerated, these results were not encouraging [24].

### Bevacizumab with chemotherapy in recurrent/metastatic HNSCC

Bevacizumab has been combined with chemotherapy in clinical trials (Table 3). In a phase II trial in the first-line treatment of recurrent/metastatic HNSCC that enrolled 40 patients, bevacizumab was combined with the antimetabolite pemetrexed [25]. This regimen resulted in a median time to progression of 5 months, median OS of 11.3 months and an overall response rate of 30%. Fifteen percent of patients had grade 3–5 bleeding events but otherwise the regimen was well tolerated. These efficacy results were comparable to historical controls using the 3-drug standard “EXTREME” regimen (platinum, 5-FU and cetuximab) [35].

**Table 3:** Bevacizumab-containing combination therapies in HNSCC

AGENTS COMBINED WITH BEVACIZUMAB	STUDY DESIGN/PHASE	#of PATIENTS	PRIMARY EFFICACY ENDPOINT	AUTHOR
**Chemotherapy**				
Pemetrexed + bevacizumab	Single arm, Phase II	*n* = 47	Median TTP 5 months	Argiris, 2011 [25]
Chemotherapy +/− bevacizumab (chemotherapy was investigator’s choice: cisplatin+ 5-FU, cisplatin+docetaxel, carboplatin+ 5-FU, or carboplatin+ docetaxel)	Randomized, Phase III	*n* = 403	Median overall survival 12.6 months with bevacizumab and 11 months without bevacizumab (*p* = 0.13)	Argiris, 2019 [26]
**Chemoradiotherapy**				
RT + docetaxel + bevacizumab	Single arm, Phase II	n = 30	3-year PFS 62%	Yao, 2015 [27]
RT + cisplatin + bevacizumab	Single arm, Phase II	*n* = 42	2-year PFS 76%	Fury, 2012 [28]
RT + 5-FU, hydroxyurea + bevacizumab	Randomized, Phase II	*n* = 26	2-year OS 58% with bevacizumab and 89% without bevacizumab	Salama, 2011 [29]
**EGFR inhibitor**				
Cetuximab + bevacizumab	Single arm, Phase II	*n* = 46	RR 16%	Argiris, 2013 [30]
Erlotinib + bevacizumab	Single arm, Phase I/II	Phase I *n* = 10 Phase II n = 48	Median PFS 4.1 months	Cohen, 2009 [31]
**Chemoradiotherapy + EGFR inhibitor**				
RT + pemetrexed+ cetuximab +/− bevacizumab	Randomized, Phase II	*n* = 78	2-year PFS 75% with bevacizumab and 79% without bevacizumab	Argiris, 2016 [32]
RT + cisplatin+ cetuximab + bevacizumab	Single arm, Phase II	n = 30	2-year PFS 88.5%	Fury, 2016 [33]
Paclitaxel+ carboplatin+ 5-FU + bevacizumab, followed by RT + paclitaxel + erlotinib + bevacizumab	Single arm, Phase II	*n* = 60	3-year PFS 71%	Hainsworth, 2011 [34]

*RT* radiotherapy, *RR* overall response rate, *OS* overall survival, *PFS* progression-free survival, *TTP* time to progression

A phase III randomized clinical trial (E1305) of bevacizumab in patients with recurrent/metastatic HNSCC was conducted by the ECOG-ACRIN Cancer Research Group [26]. The E1305 trial randomly assigned patients to receive an investigator’s-choice platinum doublet (cisplatin or carboplatin plus either docetaxel or 5-fluorouracil) with or without the addition of bevacizumab. Patients in this study had not received therapy for recurrent/metastatic disease but prior chemotherapy or cetuximab was allowed if given in the setting of prior potentially curative treatment with an interval of at least 4 months. In a total of 403 patients accrued, the median OS was 12.6 months with bevacizumab and chemotherapy versus 11 months with chemotherapy alone, a difference that was not statistically significant (HR 0.87, 95% CI 0.70–1.0, *p* = 0.22). Although the primary endpoint of the study was not met, there was a numerical survival advantage at 2, 3 and 4 years in the bevacizumab arm (25.2% vs 18.1% at 2 years, 16.4% vs 10% at 3 years, and 11.8% vs 6.4% at 4 years for chemotherapy plus bevacizumab versus chemotherapy alone). Moreover, median PFS improved from 4.3 months to 6.0 months with the addition of bevacizumab to chemotherapy (HR 0.71; *p* = 0.0012), and the overall response rate increased from 24.5 to 35.5% (*p* = 0.013). However, patients experienced more treatment-related toxicities with bevacizumab, particularly grade 3–5 bleeding. While this study provides evidence of improved antitumor activity with the addition of an anti-angiogenic agent to chemotherapy, randomized data showing survival benefit with this approach are still lacking. The study of better-tolerated anti-angiogenic agents in combination with chemotherapy or other targeted agents is warranted. Better patient selection on the basis of molecular biomarkers may contribute to optimal results.

### Anti-angiogenics with EGFR inhibitors in recurrent/metastatic HNSCC

EGFR blockade has also been a desired therapeutic target due to the proposed mechanism of preventing VEGF escape [36]. Cetuximab, a monoclonal antibody against EGFR, has been approved by the FDA for the treatment of HNSCC as monotherapy and in combination with radiotherapy or chemotherapy [35, 37, 38]. Cetuximab has been studied in combination with bevacizumab in many clinical trials. Preclinical data supported the clinical evaluation of this combination in HNSCC [30]. Argiris et al. conducted a phase II trial that investigated cetuximab plus bevacizumab in recurrent/metastatic HNSCC. Up to 1 prior regimen for recurrent/metastatic disease was allowed. The response rate of 16%, median PFS of 2.8 months and the median OS of 7.5 months appeared promising [30]. Another trial by Cohen et al. evaluated the combination of an EGFR TKI, erlotinib and bevacizumab in a similar patient population. They reported a response rate of 15%, median PFS of 4.1 months, and median OS of 7.1 months [31]. Both trials reported an acceptable rate of bleeding events (grade 3–5 bleeding of 4–5%). A previously mentioned phase II randomized trial failed to show improved efficacy with the addition of sorafenib to cetuximab [21]. A more recent phase Ib trial reported promising results with pazopanib and cetuximab in patients with recurrent/metastatic HNSCC [39]. However, appropriately designed randomized trials would be required to fully assess the potential role of these combination treatments in HNSCC.

### Bevacizumab with chemoradiotherapy or chemoradiotherapy plus EGFR inhibitors

Radiotherapy and chemotherapy are speculated to work synergistically with anti-angiogenics [40]. Therefore, anti-angiogenic combinations with chemoradiotherapy have been tested in clinical trials (Table 3). A phase I trial evaluated the combination of bevacizumab with 5-FU, hydroxyurea, and radiotherapy for patients with recurrent or poor prognosis HNSCC with a goal of finding the maximum tolerated dose of bevacizumab [41]. Two-thirds of the patients had been previously irradiated. Investigators determined that the adverse effects in this patient subset were comparable to historically re-irradiated controls. With a median OS for re-irradiated patients of 10.3 months, the antitumor effect was sufficient to support further interest in combinations of bevacizumab with radiotherapy. However, a subsequent phase II randomized trial in locally advanced HNSCC with twice-daily radiotherapy, hydroxyurea, 5-FU with or without bevacizumab was terminated due to unexpectedly higher locoregional progression rate and worse two-year survival in the bevacizumab arm [29].

The incorporation of bevacizumab into a more widely used platinum-based chemoradiotherapy regimen was also tested. In a phase II trial bevacizumab was added to concurrent chemoradiotherapy with high-dose cisplatin given in split doses (days 1, 2, 22, 23, 43, and 44) and 70 Gy of intensity-modulated radiation therapy (IMRT) [28]. Forty-two patients with previously untreated disease (93% with an oropharyngeal primary, 45% of whom confirmed positive for human papillomavirus) were enrolled and treated. The 2-year PFS was 76% and the 2-year OS was 88% which was promising and the toxicity profile was acceptable.

A phase II trial in locally advanced HNSCC examined the addition of bevacizumab to radiotherapy and docetaxel in 30 patients (67% with an oropharyngeal primary) and reported a 3-year PFS of 62% and a 3-year OS of 68% [27], whereas another phase II trial evaluated the addition of bevacizumab to an induction chemotherapy regimen and subsequent concurrent chemoradiotherapy [34]. In the latter trial, patients (60% had an oropharyngeal primary) were treated with a novel neoadjuvant approach of 6 weeks of paclitaxel, carboplatin, 5-FU, and bevacizumab before continuing with concurrent radiotherapy with paclitaxel, bevacizumab, and erlotinib. The 2-year and 3-year PFS was 83 and 71%, respectively, and the 2-year and 3-year OS was 90 and 82%, respectively, and no unanticipated toxicities were seen [34].

Other trials have looked into the incorporation of both bevacizumab and cetuximab in combined modality treatment. A single arm phase II trial of the combination of radiotherapy, cisplatin, cetuximab and bevacizumab that enrolled 30 patients (24 with an oropharyngeal primary, of whom 71% had human papillomavirus-associated tumors) reported a promising PFS of 88.5% at 2 years [33]. It is difficult to evaluate the results of single arm studies in locally advanced HNSCC and make comparisons with historical controls because of many potential biases and the variability in inclusion of good prognosis patients (e.g. those with human papillomavirus positive oropharyngeal tumors). A phase II randomized trial that evaluated the addition of bevacizumab in a combined modality regimen in 78 patients with previously untreated locally advanced HNSCC was conducted by Argiris et al [32] Patients received IMRT 70 Gy with concurrent cetuximab and pemetrexed, with or without bevacizumab. The two-year PFS was not different with or without bevacizumab (75% versus 79%). Moreover, this study demonstrated increased toxicities in patients treated with bevacizumab [32].

Overall, current evidence does not support added benefit from the combination of anti-angiogenics and EGFR-inhibitors in locally advanced HNSCC. Further evaluation of strategies incorporating anti-angiogenenic targets with radiotherapy may be appropriate. Nevertheless, the data so far do not justify the conduct of a phase III trial of anti-angiogenics in locally advanced HNSCC.

### Anti-angiogenics with immune checkpoint inhibitors

Anti-angiogenics in combination with immune checkpoint inhibitors have established benefit in multiple tumor types [42, 43]. Additionally, the immune checkpoint inhibitors nivolumab and pembrolizumab have both been FDA approved for the treatment of recurrent/metastatic HNSCC. The advantage of combining one of these agents with an anti-angiogenic agent remains unknown in HNSCC, however, it could potentially potentiate a synergistic effect, leading to greater antitumor effect. Mechanistically, anti-angiogenic therapies are thought to promote an immunosupportive environment by increasing the trafficking of T cells into tumors and reduce immunosuppressive responses, thereby overcoming resistance to checkpoint inhibitors [44]. Therefore, several ongoing clinical trials are evaluating simultaneous targeting of angiogenesis and tumor immunity. A phase Ia/b multicohort trial studied ramucirumab with the programmed cell death protein 1 (PD-1) inhibitor pembrolizumab with good safety and preliminary efficacy results [45]. A phase Ib/II trial of lenvatinib plus pembrolizumab in patients with multiple solid tumor types, including HNSCC, demonstrated promising preliminary clinical activity in 22 patients with recurrent/metastatic HNSCC (response rate of 46% and median PFS of 4.7 months) with expected toxicities [46]. Similar combination strategies are being actively pursued, including ongoing phase II trials of ramucirumab plus pembrolizumab and bevacizumab plus atezolizumab in recurrent/metastatic HNSCC (NCT03650764, NCT03818061).

## Conclusion

The induction and sustainment of angiogenesis is one of the hallmarks of solid tumors. Various pro-angiogenic factors have been found to be upregulated in HNSCC, correlating with more aggressive disease. Anti-angiogenic agents with differing mechanisms of action have been tested in the treatment of HNSCC with mixed success. The field is open to further investigations of combinations of anti-angiogenic agents with immunotherapeutic, molecularly targeted and chemotherapeutic agents in HNSCC. In the future, individualized anti-angiogenic treatment based on molecular tumor characterization will likely lead to improved outcomes.

## Data Availability

Data sharing not applicable to this article as no datasets were generated or analyzed during the current study.

## Abbreviations

ALK1-Fc: Activin receptor-like kinase 1 fused to a human Fc domain
EGFR: Epidermal growth factor receptor
FGFR: Fibroblast growth factor receptor
HNSCC: Head and neck squamous cell carcinoma
IMRT: Intensity-modulated radiation therapy
NSCLC: Non-small cell lung cancer
ORR: Overall response rate
OS: Overall survival
PD-1: Programmed cell death protein 1
PDGFR: Platelet-derived growth factor receptors
PFS: Progression-free survival
PS: Performance status
TKI: Tyrosine kinase inhibitor
TTP: Time to progression
VEGFR: Vascular endothelial growth factor receptor
